# Heart Transplant Rejection: From the Endomyocardial Biopsy to Gene Expression Profiling

**DOI:** 10.3390/biomedicines12081926

**Published:** 2024-08-22

**Authors:** Anca Otilia Farcas, Mihai Ciprian Stoica, Ioana Maria Maier, Adrian Cornel Maier, Anca Ileana Sin

**Affiliations:** 1 Doctoral School of Medicine and Pharmacy, George Emil Palade University of Medicine, Pharmacy, Sciences and Technology of Targu Mures, 540142 Targu Mures, Romania; ancacontac@gmail.com; 2 Department of Cell Biology, George Emil Palade University of Medicine, Pharmacy, Sciences and Technology of Targu Mures, 540139 Targu Mures, Romania; ileana.sin@umfst.ro; 3 Department of Nephrology/Internal Medicine, Mures County Clinical Hospital, 540103 Targu Mures, Romania; 4 Department of Internal Medicine, George Emil Palade University of Medicine, Pharmacy, Sciences and Technology of Targu Mures, 540139 Targu Mures, Romania; 5 Emergency Military Hospital, 800150 Galati, Romania; mioanamaier@gmail.com; 6 Faculty of Medicine and Pharmacy, Dunarea de Jos University, 800008 Galati, Romania; 7 Department of Pathology, Clinical County Emergency Hospital, 540136 Targu Mures, Romania

**Keywords:** heart transplant (HT), acute cellular rejection (ACR), antibody-mediated rejection (AMR), endomyocardial biopsy (EMB), donor-derived cell-free DNA (dd-cfDNA), donor-specific antibody (DSA), gene expression profiling (GEP), treatment

## Abstract

Heart transplant prolongs life for patients with end-stage heart failure but rejection remains a complication that reduces long-term survival. The aim is to provide a comprehensive overview of the current status in HT rejection. EMB is an invasive diagnostic tool, consisting in the sampling of a fragment of myocardial tissue from the right ventricular septum using fluoroscopic guidance. This tissue can later be subjected to histopathological, immunohistochemical or molecular analysis, providing valuable information for cardiac allograft rejection, but this procedure is not without complications. To increase the accuracy of the rejection diagnosis, EMB requires a systematic evaluation of endocardium, myocardium, interstitium and intramural vessels. There are three types of rejection: hyperacute, acute or chronic, diagnosed by the histopathological evaluation of EMB as well as by new diagnostic methods such as DSA, ddcfDNA and gene expression profiling, the last having a high negative predictive value. More than 50 years after the introduction of EMB in medical practice, it still remains the “gold standard” in monitoring rejection in HT recipients but other new, less invasive diagnostic methods reduce the number of EMBs required.

## 1. Introduction

Heart transplantation stands as the ultimate treatment for end-stage heart failure, offering a chance at extended life and improved quality of life for patients. However, despite advancements in surgical techniques, immunosuppressive therapies and post-transplant care, rejection remains a significant challenge. Rejection occurs when the recipient’s immune system recognizes the transplanted heart as foreign and mounts an immune response against it. Understanding the mechanisms underlying heart transplant rejection is crucial for developing strategies to prevent and manage this complication effectively. 

Unlike kidney or liver transplantation, in the case of graft failure after heart transplantation, the patient’s death occurs immediately in the absence of a new transplant or mechanical circulatory support (MCS) devices. Therefore, the identification of cardiac allograft rejection is essential in the monitoring of heart transplant recipients, regardless of whether it is immune-mediated rejection, specifically, acute cellular rejection (ACR), antibody-mediated rejection (AMR) or cardiac allograft vasculopathy (CAV).

In this review we will focus to provide a comprehensive overview of the current understanding of heart transplant rejection, including its immunological basis, risk factors, diagnostic methods and treatment strategies.

## 2. Historical Consideration

From the first transplant performed by Christian Barnard in December of 1967 to over 100 HTx performed worldwide in the next year, interest in this procedure rapidly declined because the poor survival rate became obvious. The main problem, then and now, was the prevention and control of cardiac allograft rejection. In that early era of cardiac transplantation, corticosteroids and azathioprine were among the few therapeutic agents used in rejection, followed by the introduction of rabbit antithymocyte globulin (ATG) that increased the survival rates. The discovery of cyclosporine by Calne [1] and its insertion into clinical use in the late 1970s [2] has played a major role due to the selective immunoregulation of T cells. A few years later, another molecule called FK 506 [3] appeared in the list of immunosuppressive drugs; following that, another category of immunosuppressive drugs represented by proliferation signal inhibitors (PSI), including Rapamycin (Rapa) and Everolimus (EvE), would be introduced in immunosuppressive therapy in organ transplantation. The most used methods for detecting rejection involved examining ECG changes (decrease in QRS voltage [4], arrhythmias or changes in the T wave or QT interval), but in 1973, Caves [5] proposed endomyocardial biopsy (EMB) as a method for monitoring heart transplant recipients. The first bioptome used for endomyocardial biopsies was introduced in clinical practice by Sakakibara and Konno in 1962. In the following decade, the Stanford group developed a flexible bioptome, considerably reducing the risks associated with this interventional procedure. During that period, the popularity of EMB increased significantly, being accepted all over the world, so that it became imperative to create some rejection classification systems. In 1990, the International Society for Heart and Lung Transplantation (ISHLT) [6] realized the first unique classification for cardiac allograft rejection, which was later replaced by the second ISHLT classification, in 2004 [7].

## 3. Endomyocardial Biopsy—“Almost Everything” about It

### 3.1. Endomyocardial Biopsy Technique

Masson [8] was one of the first to modify the endomyocardial biopsy technique proposed by Caves, and in 1974, the method was adopted as a standard protocol for monitoring heart transplant patients at Stanford University.

The transvascular approach using a bioptome (Konno–Sakakibara bioptome) was used for the first time in Japan, at the beginning of the 1960s. Ten years later, a flexible bioptome that could perform serial endomyocardial biopsies was developed by the Stanford group [9]. Since then, the technique has been refined, being unanimously accepted throughout the world. 

EMB is an invasive diagnostic tool, consisting of the sampling of a small fragment of myocardial tissue, typically from the right ventricular septum, using fluoroscopic guidance. The tissue thus obtained can later be subjected to histopathological, immunohistochemical or molecular analysis, providing valuable information for cardiac allograft rejection.

Different venous approaches are used to perform an endomyocardial biopsy: transjugular or transfemoral, for endomyocardial biopsies from the right ventricle; and via femoral or radial artery, for biopsies from the left ventricle [10]. 

**Figure 1:**
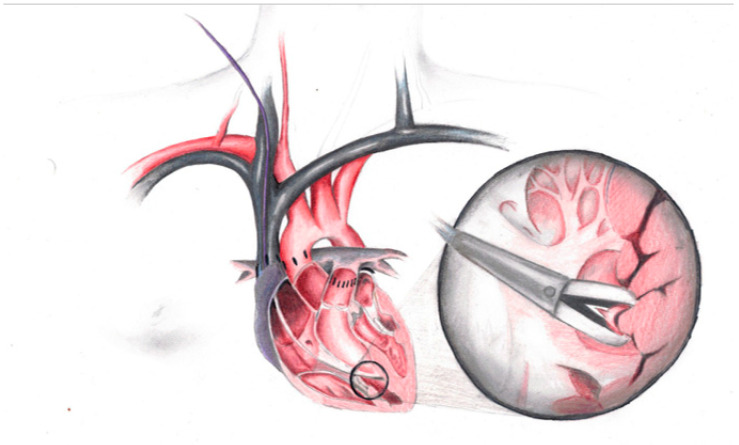
Artistic representation of EMB technique.

Under fluoroscopic or echocardiographic [11] guidance and the monitoring of heart rate, oxygen saturation and non-invasive blood pressure, a bioptome is introduced most frequently in the internal jugular vein and is advanced through a sheath that previously has been placed using the standard Seldinger technique. This procedure takes place in a cardiac catheterization room when fluoroscopic guidance is used. However rarely, under transesophageal echocardiographic control, EMB can be performed at the patient’s bedside [12]. The bioptome must be positioned open along the interventricular septum and remain open until it touches the ventricular wall. The next step is closing the bioptome and then withdrawing it, followed by removing the bioptome from the sheath and flushing the sheath. This sequence of steps can be repeated three–five times, until the desired number of tissue fragments are obtained (Figure 1).

### 3.2. Complications of EMB

Although they are rare, EMB complications can occur both during the procedure or in the immediate post-procedure period and in the long term. Therefore, they can be systematically divided into complications related to vascular access and complications related to the biopsy procedure. 

Among the complications associated with the vascular approach, in a study carried out on 2454 biopsies, C Baraldi-Junkins et al. [13] report an incidence of 2.3% complications associated with catheter insertion, including carotid puncture (1.8%), vasovagal reaction (0.1%) and prolonged bleeding (0.4%). Rossana Bussani et al. [14] mention a 1% incidence of major complications during EMB even when this is performed by teams with extensive experience. Among the major complications that can occur during endomyocardial biopsy, the following are mentioned in the literature: vasovagal syncope, ventricular perforation, pericardial tamponade, heart block, supraventricular and ventricular arrhythmias, accidental arterial puncture, pneumothorax, vascular damage, nerve damage, pulmonary embolism, coronary-cameral fistula formation [15], bleeding complications and damage to the tricuspid valve, especially in patients undergoing repeated biopsies [16], as in the case of heart transplanted patients. Other complication that may occur are nerve block/injury, hematoma, pericardial effusion, arteriovenous fistula and deep vein thrombosis [11].

There are numerous studies, among which, we mention those conducted by Saraiva et al. [17] and Baraldi-Junkins C et al. [13], who both found a very low rate of complication produced by EMB, between 0.5–1% (Saraiva) and 3% (Baraldi-Junkins) [13,18,19].

### 3.3. Histopathological Examination of the EMB

Throughout history, numerous systems have been used to grade the rejection phenomenon, but in time, the classification of Margaret E. Billingham, Stanford University and Texas Heart Institute, was imposed in the USA, while in Europe, the most used one was the Hannover classification. 

In 1990, ISHLT [6] proposed a unique classification of heart allograft rejection, which was used for 15 years, until 2004, when it was replaced by another ISHLT classification [7]. A schematic comparison between the two ISHLT classifications of ACR is shown in Table 1.

To increase the accuracy of the rejection diagnosis, each endomyocardial biopsy requires a systematic evaluation of both the endocardium and myocardium, as well as the interstitium and intramural vessels.

In order to correctly evaluate the myocardium, we will provide a brief reminder of its normal histology. Usually, we will visualize cardiomyocytes as elongated, branched cells, having a central nucleus, often containing lipofuscin around it. Cardiomyocytes are connected among themselves by junctions called intercalated discs. Among the pathological changes, on usual HE staining, the following changes are noticeable: hypertrophy with nuclear polyploidy often found in hypertrophic myocytes, atrophy, cytoplasmic vacuolization, myocytolysis, coagulative necrosis and architectural disorganization. 

The interstitium is, perhaps, the most important in terms of the information it can provide in the evaluation of EMB. From this level, information can be extracted about both cellular components (fibroblasts, histiocytes, smooth muscle cells, myofibroblasts, mast cells and adipocytes) and inflammatory infiltrates (lymphocytes, eosinophils and neutrophils) and the response they generate. Currently, there is the possibility of immunophenotyping if it is necessary to characterize the lymphocyte population. The suspicion of a post-transplant lymphoproliferative disorder or a lymphoma should be raised in the cases of evidence of atypia. However, lymphomas are rare in adult recipients (1–2% by 5–10 years post-transplant) [20].

Intramural vessels undergo changes during episodes of acute cellular rejection and humoral rejection, but also during chronic rejection (CAV). In AMR (antibody mediated rejection), histopathological changes appear in the capillary beds that include endothelial swelling or denudation, the presence of macrophages or neutrophils in capillaries, interstitial edema, congestion and hemorrhage, in severe cases. Sometimes fibrin may also be found in the vessel beds. Immunopathologic findings include the deposition of IgG, M or A, and positive staining for components of the complement cascade [21].

Special staining methods such as Masson trichrome, Weigert Van Gieson, PAS, Gram, Gomori or immunohistochemical staining for CD31, CD34, CD45, CD68, C4d, etc., are among the most helpful tools in the diagnosis of rejection.

## 4. Rejection in Heart Transplant

### 4.1. Immunological Basis in Heart Transplant Rejection

In order to effectively treat rejection episodes, it is absolutely necessary to understand the pathogenesis and immunological basis of rejection and the action mechanism of immunosuppressive drugs. Two essential components are involved in the immune response: a cellular one (lymphocytes, macrophages, mast cells, antigen-presenting cells (APC), eosinophils, basophils and neutrophils) and a molecular one comprising antigens, major histocompatibility complex (MHC), cytokines, adhesion molecules, receptors, enzymes, immunoglobulins and the complement with its fractions. MCH is encoded by more than 200 genes located on the short arm of chromosome 6 [22], characterized by a marked polymorphism and a high allelic variability, thus leading to very different responses within the rejection phenomenon [23]. 

MCH antigens are divided into several classes: Class I (with subclasses HLA-A, HLA-B and HLA-C), class II (with subclasses DR, DQ and DP, which are normally found in APC and endothelial cells) and Class III (which includes molecules that are not directly involved in antigen presentation but are involved in humoral immunity). In heart transplantation, as well as in the transplantation of other solid organs, two MHC classes have a crucial role: MHC class I (HLA-A, HLA-B and HLA-C) and MHC class II (DR, DQ and DP) [24,25].

In essence, the rejection is due to the incongruity between the donor’s MHC amino acids and the recipient’s MHC amino acids, so that even the variability of a single amino acid causes a rejection reaction. There are studies [22] that have proven that only 1–10% of the total T lymphocytes of an individual show alloreactivity (the response of an individual’s T lymphocytes through an activation, proliferation and differentiation reaction to the interaction with the cells of another individual, unrelated, from the same species) by recognizing the polymorphism allelic from the level of the MHC, thus leading to an immediate strong immune response.

**Figure 2:**
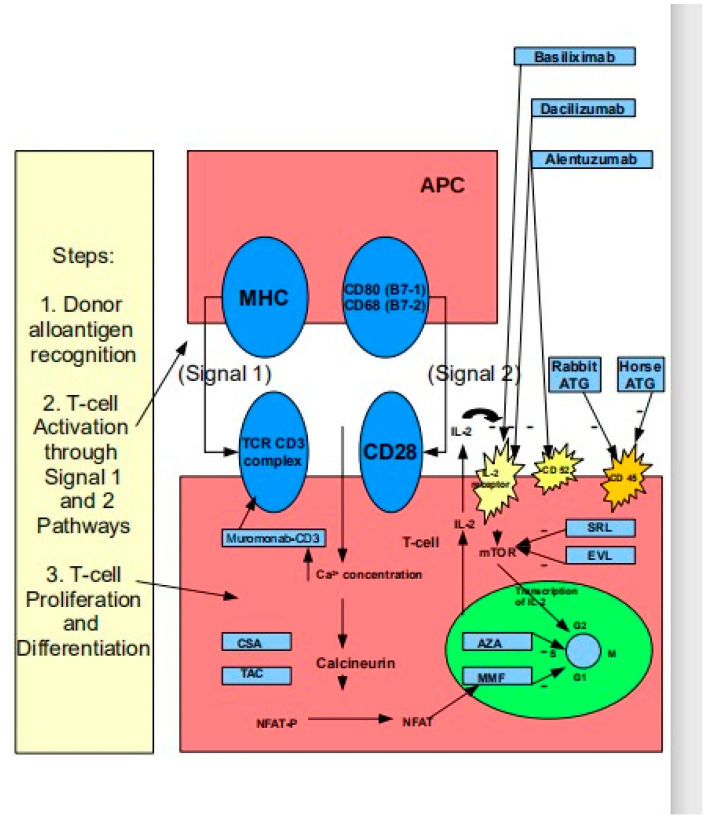
Schematic graphic representation of the immunological basis of rejection.

The NFAT (nuclear factor of activated T-cells) is dephosphorylated by activated- calcineurin, which, in turn, is activated by the increased concentration of Ca^2+^ via the signal 1 and signal 2 pathways. Signal 1 is initiated by the interaction of the T cell receptor (TCR) with its ligand, whereas signal 2 is initiated by an interaction between costimulatory molecules on the antigen-presenting cell (APC) and counterreceptors on the T cell [26]. IL-2 transcription, initiated by NFAT entrance in the nucleus, leads to the former exit from here via an autocrine pathway, after which it immediately binds to the IL-2 receptor on the surface of the T-cell. This binding between IL-2 and its receptor leads to mTOR (mammalian target of rapamycin) activation, which will bring with it the proliferation and differentiation of T lymphocytes [27]. A schematic graphic representation of the immunological basis of rejection is shown in Figure 2.

### 4.2. Types of Rejection and Risk Factors

According to the time it takes to appear, the rejection can be classified into three types: hyperacute, occurring within minutes or hours following the heart transplant, leading to graft dysfunction and, most often, graft loss within 24 h; acute, which, in turn, is divided into acute cellular rejection (ACR) and antibody-mediated rejection (AMR); and chronic, also known as cardiac allograft vasculopathy (CAV). 

Hyperacute rejection is characterized histopathologically by vascular congestion, thrombosis and hyaline microthrombi, vasculitis with fibrinoid necrosis of small and medium vessels, along with edema and interstitial granulocytic inflammatory infiltrate. This unfortunate situation occurs if there are preformed antibodies (PRA) against donor antigens and more frequently affects patients who have received multiple blood transfusions and women with multiple pregnancies. However, for candidates with high PRA levels, desensitization protocols are used for pretreatment, including plasmapheresis, intravenous immunoglobulin (IVIG), rituximab (chimeric monoclonal anti-CD20 antibody) and bortezomib (proteasome inhibitor), considerably reducing the risk of developing hyperacute rejection [28].

Acute cellular rejection (ACR) or T-cell mediated rejection, was promptly described in the early 1960s and remains the target of most current maintenance immunosuppression agents. It represent lymphocytic infiltration of the perivascular and interstitial myocardial compartments leading to myocyte damage and necrosis, altered myocardial architectural appearance and allograft dysfunction, causing 9% of deaths between the first and the third year post-transplant [29]. The highest frequency of ACR is found in the first 3–6 months after transplantation, with ISHLT reporting a percentage of 19% of all HT patients in their registries between 2004 and 2010 that required at least one treatment for acute rejection (ACR or AMR) [30].

There are two different pathways for the recognition of alloantigens: the direct pathway, in which T cells “directly” recognize intact non-self MHC molecules present on the surface of donor cells; and the indirect pathway, which involves the ability of T-lymphocytes to recognize the donor’s MHC molecules processed by APC. The MHC molecules located on the surface of the APC present in their grooves peptides that interact with the TCR, CD8+ lymhocytes having the role to recognize peptide/MHC Class I complexes and CD4+ lymphocytes being involved in the recognition of peptide/MHC Class II complexes. In addition to MHC molecules, there are also minor histocompatibility antigens that can induce a CD4 or CD8 lymphocytes response [31]. In this way, the T-lymphocytes mediate an inflammatory response against the allograft, thus leading to myocyte necrosis and graft failure [32].

Histopathologically, ACR is characterized by the presence of inflammatory infiltrate, predominantly comprised of lymphocytes, macrophages and, in some cases, eosinophils. If the neutrophils are present, the suspicion of a different process such as infection, healing ischemic injury or AMR occurs. However, in severe cases of ACR, neutrophils may also be present. Plasma cells are characteristic of lymphoproliferative disorders, the Quilty effect or healing ischemic injury.

Antibody mediated rejection (AMR), also known as humoral rejection or B-cell mediated rejection, of the cardiac allograft was first clinically described in the late 1980s by Herskowitz et al. [33], and followed soon thereafter by pathologic evidence to support a unique rejection process independently from cellular mechanisms [34]. B-cells originate in bone marrow as common lymphoid progenitor cells, which become pro-B cells, and next, pre-B cells, and finally, immature B-cells. Immature B-cells leave the bone marrow and migrate into the lymph nodes and spleen to complete the process of maturation. Plasma cells (activated mature B-cells) produce antibodies that are specific for each individual antigen such as proteins expressed on the cell surface of a transplanted heart. This immune activation by antibodies damages the allograft via complement cascade-mediated fixation and activation, which injures the allograft and also acts as a biochemical “amplifier”, signaling other parts of the innate and adaptive immune systems such as neutophils, pro-inflammatory molecules and cytokines.

There are some risk factors for developing AMR, among which, we list situations that expose the patient to human products: blood transfusion or blood product administration, pregnancies, repeat transplantation and, specific to heart transplantation, the use of extracorporeal and intracorporeal mechanical circulatory support devices such as left ventricular assist systems (LVAS), bi-ventricular assist devices, total artificial hearts, extracorporeal membranous oxygenators (ECMO) or intra-aortic counterpulsators [35]. Other potential risk factors for developing AMR mentioned in the literature include female sex [36], elevated preformed antibodies [37], cytomegalovirus (CMV) seropositivity, a positive crossmatch [38,39] and prior sensitization to OKT3 [40].

Among the signs and symptoms indicative of acute rejection mentioned are dyspnea, edema, fatigue, nausea, fever and arrhythmias.

When the goal is to identify the histopathological changes in AMR, attention must be directed to the capillary beds because the changes appear almost exclusively at this level. Endothelial swelling or denudation, the presence of macrophages or neutrophils in capillaries, interstitial edema, congestion and, potentially, hemorrhage, in severe cases, are common findings. Sometimes, fibrin may also be found in the vessel beds. Immunopathological findings include the deposition of IgG, M or A and positive staining for components of the complement cascade.

In 2013, ISHLT revised the 1990 working formulation for the standardization of nomenclature in the diagnosis of AMR and the changes are summarized in Table 2 [7].

The chronic form of rejection following heart transplantation is represented by cardiac allograft vasculopathy (CAV), which remains one of the dreaded long-term complications of HT due to the high death rate, with ISHLT mentioning a death rate of 32% between the 5th and 10th year after transplantation. Although the incidence of CAV has decreased significantly due to therapeutic strategies to prevent it, approximately 30% of patient with HT have CAV after 5 years, and the percentage reaches almost 50% at 10 years after transplant [20].

Over the time, immunological and non-immunological risk factors of CAV have been highlighted. Out of the non-immunological risk factors, we list the following: old age of donor, ischaemia–reperfusion lesions, recipient hypertension, diabetes mellitus or hypercholesterolemia and CMV infection. Even if there are numerous non-immunological factors involved in CAV, the latter is considered an immunological disease, being favored by the presence of alloantibody and occurrence of acute rejection [41,42]. As Ticehurst et al. mention, the presence of de novo DSAs (donor specific antigens) against anti–human leukocyte antigen (HLA) Class II, especially DQ, presents an increased risk for developing CAV [43].

Atherosclerosis is similar to CAV in that both diseases affect intima and adventitia, but the lesions produced by atherosclerosis differ from those of CAV regarding cellular and extracellular composition as well as the limited location, eccentricity and focality of atheromas [44]. CAV is characterized by a thickening of the coronary wall, different from classic atherosclerosis [45]. Intimal thickening in CAV leads to narrowing of the arterial lumen and consists of two layers: the original, thickened intimal layer, made up of smooth muscle fibers adjacent to the internal elastic lamina; and an additional (new) internal layer, located at the level of the luminal part consisting of loose connective tissue, smooth muscle fibers and inflammatory infiltrate consisting of mononuclear cells [46].

## 5. New Diagnostic Methods in Acute Rejection

Although EMB has proven useful for over 50 years in the diagnosis of rejection, new, less invasive diagnostic methods are to be expected so that these could be considered the new “gold standard”. With the evolution of technology, the concern for the discovery of non-invasive diagnostic methods has become increasingly intense; therefore, methods including micro-RNA (mi-RNA), donor specific antibodies (DSAs), donor-derived cell-free DNA (ddcfDNA) and gene expression profiling (GEP) are now available for the diagnosis of rejection [47,48,49].

Donor specific antibodies (DSAs) can be identified utilizing solid-phase assays consisting of beads conjugated with single or multiple HLA antigens [50]. These beads are coated with fluorescein-labeled antigens, which emit fluorescent light in the presence of the known HLA antibody. The degree of fluorescence is directly proportional to the circulating amount of the involved HLA antibody.

DSAs were included by ISHLT in 2018 [51], in post-transplant surveillance guidelines, with the recommendation for them to be performed at 1, 3, 6 and 12 months after transplantation due to DSAs association with AMR, CAV (cardiac allograft vasculopathy) and graft dysfunction [52,53]. However, most patients present HLA antibodies post-transplant, but only patients with persistent DSAs are affected by worse survival rates [54]. In a study in which researchers analyzed the presence of DSAs every year after transplantation up to 13 years post-transplant, in a group of 243 heart transplant recipients, Smith J.D. et al. [55] reported the presence of de novo DSA in 57/243 patients (23,45%), this predicting a poor survival rate (hazard ratio (HR) = 3.067). Another interesting aspect reported in the previously mentioned study is the fact that among the patients who died from CAV/AR, 57% had DSA, while only 19% of the patients who died from CAV/AR did not have DSA [55].

The principle on which donor-derived cell-free DNA (ddcfDNA) is based is that, with the destruction of the cardiomyocytes of the allograft, small fragments of DNA are released, which end up in the recipient’s circulation. In the study conducted by De Vlaminck [56], in which 65 heart transplant patients were included, and monitored by both EMB and ddcfDNA for the diagnosis of acute rejection episodes, 356 EMB and 565 plasma samples were analyzed to quantify the performance of ddcfDNA in the detection of acute rejection. It reports that the sensitivity and specificity of ddcfDNA are comparable to those of the EMB performance. A significant increase in the level of ddcfDNA in the recipient’s circulation is frequently encountered postoperatively, but the level decreases to 0.13% at 28 days post-transplantation [57], but other studies have shown that ddcfDNA stabilizes at a baseline level 1 week post-transplantation [58,59]. With a high negative predictive value, the level of ddcfDNA correlates with the grade of acute rejection, this being elevated during rejection episodes and lessening after treatment [57,60]. There are other situations in which high levels of ddcfDNA can be found, such as myocardial ischemia, myocardial trauma, pregnancy and multiorgan transplant [61], which requires the careful interpretation of these tests. On the other hand, researchers such as Afzal et al. [62] report, in their study, that in over 20% of the cases included in the study (35/106), the serum level of ddcfDNA was high, without the patients showing clinical signs of rejection. Another disadvantage of this diagnostic method may be the impossibility of distinguishing between AMC and ACR. Although the results of the current studies are promising, additional data are needed to confirm the usefulness of implementing this diagnostic method in the monitoring of heart transplant patients.

Gene expression profiling (GEP) can represent a useful diagnostic tool in the case of transplanted patients, as there are clinical trials like IMAGE [63] that have confirmed its usefulness, with ISHLT [64] including it in the guidelines for highlighting the absence of cellular rejection. A major limitation of this diagnostic method is the impossibility of detecting AMR.

A test that evaluates 20 genes, 11 of which are related to rejection, is now available and is included in the ISHLT recommendations. The AlloMap^®^ test results are represented by a score between 0 and 40, indicating the probability of ACR. In a study evaluating the impact of CMV infection on the results of the AlloMap^®^ test, a significant increase in the score was detected in patients infected with CMV, even in the case of asymptomatic viremia, compared to those without CMV infection present [65].

Combining the two diagnostic methods presented above leads to superior results compared to the individual use of one of them [66].

## 6. Treatment Strategies

### 6.1. Pharmacotherapy of Immunosuppressive Drugs in Heart Transplantation

Immunosuppressive drugs are critical in the management of heart transplant patients to prevent acute and chronic rejection of the transplanted organ. The pharmacotherapy of these drugs involves multiple classes that act through various physiological mechanisms to achieve immunosuppression. Here are the primary classes of drugs used in immunosuppression and their mechanisms:

**Calcineurin Inhibitors (CNIs)**

Physiology and Mechanism of Action: CNIs, which include drugs like cyclosporine and tacrolimus, are the cornerstone of immunosuppression in heart transplantation. These drugs work by inhibiting the calcineurin pathway, a critical step in the activation of T-lymphocytes. Normally, calcineurin dephosphorylates the nuclear factor of activated T-cells (NFAT), allowing it to translocate to the nucleus and initiate the transcription of interleukin-2 (IL-2), a crucial cytokine for T-cell proliferation. By binding to intracellular proteins (cyclophilin for Cyclosporine and FKBP-12 for Tacrolimus), CNIs prevent the activation of calcineurin, thereby blocking NFAT dephosphorylation and the subsequent production of IL-2, leading to suppressed T-cell activation and proliferation [67].

**Antiproliferative Agents**

Physiology and Mechanism of Action: This class includes mycophenolate mofetil (MMF) and azathioprine. MMF inhibits the enzyme inosine monophosphate dehydrogenase (IMPDH), which is essential for the de novo synthesis of guanosine nucleotides. T- and B-lymphocytes largely use this pathway for DNA synthesis, and its inhibition by MMF leads to a decrease in lymphocyte proliferation [68]. Azathioprine, on the other hand, is metabolized into 6-mercaptopurine, which incorporates into DNA and RNA, thereby disrupting their synthesis and impairing cell division, particularly in proliferating immune cells [69].

**mTOR Inhibitors**

Physiology and Mechanism of Action: Sirolimus (rapamycin) and everolimus are examples of mTOR inhibitors used in heart transplantation. These drugs bind to FKBP-12, forming a complex that inhibits the mechanistic target of rapamycin (mTOR). mTOR is a kinase that regulates cell growth, proliferation and survival. By inhibiting mTOR, these drugs prevent the progression of T-cells from the G1 to the S phase of the cell cycle, thus blocking their proliferation and reducing the immune response against the transplanted organ [70].

**Corticosteroids**

Physiology and Mechanism of Action: Corticosteroids such as prednisone are broad-spectrum immunosuppressants that inhibit multiple components of the immune response. They act by reducing the production of pro-inflammatory cytokines (like IL-1, IL-2, IL-6 and and TNF-α), suppressing the activation of T-cells and macrophages, and promoting the apoptosis of activated lymphocytes. These effects are mediated through the binding of corticosteroids to glucocorticoid receptors, leading to changes in gene transcription [71].

The pharmacotherapy of immunosuppressive drugs in heart transplantation is a complex and multifaceted approach involving various classes of drugs. Each class targets different pathways and mechanisms within the immune system to prevent organ rejection and ensure the long-term survival of the transplanted heart. Understanding these mechanisms is crucial for optimizing therapy and minimizing adverse effects in transplant recipients.

### 6.2. Immunosuppression after HT

As in the case of any other transplanted organ, regarding heart transplant, Chang et al. [72] mentioned some basic principles that must be respected in terms of immunosuppressive treatment:

- Immediately postoperatively, the highest levels of immunosuppression should be used followed by tapering these levels in the first year, eventually reaching the lowest maintenance levels of immunosuppression, trying to find the balance between the prevention of graft rejection and the minimization of drug toxicity.
- It is advisable to use small doses of multiple drugs with non-overlapping toxicities rather than higher doses of fewer drugs to minimize side effects.
- Excessive immunosuppression should be avoided because it heightens the risk of adverse effects, including increased susceptibility to infections and malignancy.

In heart transplant, immunosuppressive drugs can be divided into three main groups: drugs for induction, drugs for maintenance and drugs for acute rejection episodes. In the category of drugs used in the induction phase of immunosuppression, the following are mentioned:

- **Anti-thymocyte globulins** (rabbit ATG and horse ATG) that have multiple distinct antigen-combining sites resulting in the depletion of circulating T-cells and apoptosis of activated T-cells. These have strong immunosuppressive effect but also major side effects like thrombocytopenia, leukopenia and anemia.
- **Interleukin-2 receptor antagonists** whose mechanism of action is blocking the interleukin 2 (IL-2) receptor on activated T-cells.
- **Anti-CD3 antibodies** (muromonab-CD3)
- **Anti-CD52 antibodies** (alemtuzumab)

In 2022, ISHLT [73] reported the use of induction immunosuppressive medication in approximately 50% of transplant patients, using IL-2R antagonist, polyclonal ALG/ATG or alemtuzumab, induction drugs being indicated especially in patients with antibody sensitization or multi-organ transplantation and in an attempt to protect the kidneys to allow delay in calcineurin inhibitor (CNI) therapy. But it should also be taken into account that there are studies such as the one conducted by Amin AA et al. [74] that found that recipients who received induction therapy using rabbit anti-thymocyte globulin (ATG) or IL-2R antagonist therapy developed more acute rejection episodes (20.2% vs. 19%, respectively) compared to patients who did not benefit from any induction (16.8%) prior to HTx.

In the therapeutic arsenal used in the immunosuppression maintenance phase, the combinations of two or three classes of drugs are included, such as CNI plus anti-metabolites or proliferation signal inhibitors (PSI). The mechanisms of action and the main side effects of these drugs are shown in the Table 3.

Chang et al. [72] mention, in the immunosuppression maintenance protocol, the use of a triple drug regimen including TAC, mycophenolate mofetil (MMF) and corticosteroid. Similar regimes are also reported by Koboshigawa et al. [75].

**Table 3:** Drugs used in the maintenance phase of immunosuppression.

	CNI	Anti-Metabolites	Proliferation Signal Inhibitors	Corticosteroids
**Name**	Tacrolimus (TAC), cyclosporine A	mycophenolate mofetil (MMF), enteric-coated mycophenolate sodium (EC-MPS), azathioprine (AZA)	sirolimus (SIR) and everolimus (EVL)	Methylprednisolone—intravenous administration prednisone—oral administration
**Mechanism of action**	inhibit the enzyme calcineurin in T-cells	inhibit the cell cycle by inducing nucleotides depletion	inhibit enzyme mammalian target of rapamycin	inhibit the synthesis of cytokines, suppress IL-6, interferon gamma, TNF and production of IL-1
**Side effects**	lymphoma and lymphoproliferative disorders hypertension [76] hyperlipidemia nephrotoxicity infections (bacterial, viral or fungal) [77]	leukopenia gastrointestinal diseases pancytopenia hepatitis pancreatitis [24]	drug–drug interactions dyslipidemia pancytopenia delayed wound healing oral ulcers pericardial and pleural effusions	osteoporosis osteonecrosis Cushing syndrome infections hypertension skin thinning and atrophy acne mild hirsutism facial erythema impaired wound healing

### 6.3. ISHLT Recommendations for ACR Treatment

Below, we briefly reproduce the ISHLT recommendations regarding general treatment of ACR. For asymptomatic ACR grade 1R, no treatment is needed. For asymptomatic ACR grade 2R, high-dose oral corticosteroids are recommended for 3–5 days, or high-dose intravenous corticosteroids for 3 days. In case of asymptomatic ACR grade 3R, high-dose intravenous corticosteroids are recommended for 3 days, and in the case of evidence of graft dysfunction or the absence of histologic resolution, ATG must be added. For symptomatic ACR (grade 1R–3R), it is necessary to administrate high-dose of intravenous corticosteroids for 3 days, and in the case of hemodynamic compromise or no improvement after 12–24 h, ATG must be added [64].

Javier MFDM et al. [4] found, in their study, that an alarming rate of approximately 35% of heart transplant patients require renal replacement therapy and 95% develop hypertension 5 years after the transplant, the renal damage being mainly due to the use of cyclosporine A, a fact that requires its use with discernment.

## 7. Future Directions

The field of immunosuppression in heart transplantation is continuously evolving, driven by the need to improve patient outcomes and reduce the incidence of rejection and adverse effects. Emerging research is focused on advancing diagnostic methods and developing novel therapeutic strategies to enhance the precision and effectiveness of post-transplant care. Gene editing and cell therapy, next-generation immunosuppressive agents, personalized immunosuppression regimens, nanotechnology and drug delivery systems, tissue engineering and bioartificial hearts are some directions in which the future of heart transplantation could evolve.

Gene Editing and Cell Therapy

Advancements in gene editing technologies, such as CRISPR-Cas9, offer the potential to modify immune responses at the genetic level. By editing specific genes responsible for immune activation or tolerance, it may be possible to create a more favorable environment for the transplanted heart, reducing the need for lifelong immunosuppression. Additionally, the use of regulatory T-cells (Tregs) is being explored as a therapeutic strategy to enhance immune tolerance. These cells can be expanded and modified ex vivo and then reintroduced into the patient to suppress the immune response against the transplanted organ [78].

Next-Generation Immunosuppressive Agents

Research is ongoing to develop next-generation immunosuppressive agents that are more targeted and have fewer side effects. Small molecule inhibitors, monoclonal antibodies, and fusion proteins are being designed to specifically target pathways involved in the immune response without broadly suppressing the entire immune system. For instance, novel JAK inhibitors and anti-IL-6 agents are being investigated for their ability to modulate immune responses with greater precision, potentially reducing the incidence of rejection and infection [79,80].

Personalized Immunosuppression Regimens

Personalized immunosuppression regimens are another emerging area of interest for the future of heart transplant rejection. Using machine learning algorithms and artificial intelligence (AI), researchers are developing models that can predict individual responses to different immunosuppressive drugs based on genetic, clinical and immunological data. This approach aims to optimize drug selection and dosing, minimizing the risk of rejection and adverse effects while improving overall patient outcomes [81].

Nanotechnology and Drug Delivery Systems

The use of nanotechnology in drug delivery systems is being explored to enhance the efficacy and safety of immunosuppressive drugs. Nanocarriers can be engineered to deliver drugs directly to the target cells or tissues, reducing systemic exposure and minimizing side effects. Additionally, nanoparticles can be designed to release drugs in response to specific stimuli, providing controlled and sustained drug release. This technology has the potential to revolutionize the way immunosuppressive drugs are administered and could lead to more effective and safer treatment protocols [82].

Tissue Engineering and Bioartificial Hearts

Looking further into the future, tissue engineering and the development of bioartificial hearts are areas of cutting-edge research. Scientists are exploring ways to create functional heart tissues using stem cells and 3D bioprinting techniques. These bioengineered tissues could potentially replace damaged or diseased parts of the heart, reducing the need for whole organ transplants and the associated complications of immunosuppression. While still in the experimental stages, this research holds immense promise for transforming the future of heart transplantation and regenerative medicine [83].

The future of heart transplantation and immunosuppression lies in the integration of innovative technologies and personalized medicine approaches. As research progresses, these cutting-edge strategies have the potential to significantly improve the diagnosis, treatment and long-term outcomes of heart transplant patients, ultimately enhancing their quality of life and survival rates.

## 8. Conclusions

More than 50 years after the introduction of endomyocardial biopsy in medical practice, it still remains the “gold standard” in monitoring rejection in heart transplanted recipients. However, there are other new, less invasive diagnostic methods such as donor specific antibodies, donor-derived cell-free DNA and gene expression profiling, which have proven their usefulness, reducing the number of endomyocardial biopsies required.

However, in addition to episodes of acute rejection and CAV, which are feared complications of heart transplantation, there are other complications such as hypertension, renal failure and neoplasia, which are mainly due to immunosuppressive treatment leading to a high mortality rate among transplant patients. Once these major complications due to immunosuppressive treatment can be prevented, a new era of transplantation will begin.

## Figures and Tables

**Table 1:** Comparison between the two ISHLT classifications of ACR.

1990 ISHLT Classification		2004 ISHLT Classification	
Grade 0	No ACR	Grade 0	No ACR
Grade 1A	Focal, mild ACR Focal perivascular and/or interstitial infiltrate without myocyte damage	Grade 1R	Mild/low-grade ACR Interstitial and/or perivascular infiltrate with up to 1 focus of myocyte damage
Grade 1B	Diffuse, mild ACR Diffuse infiltrate without myocyte damage		
Grade 2	Focal, moderate ACR One focus of infiltrate with associated myocyte damage		
Grade 3A	Multifocal, moderate ACR One focus of infiltrate with associated myocyte damage	Grade 2R	Moderate/intermediate ACR Two or more foci of infiltrate with associated myocyte damage
Grade 3B	Diffuse, moderate ACR Diffuse infiltrate with myocyte damage	Grade 3R	Severe/high-grade ACR Diffuse infiltrate with multifocal myocyte damage, ±edema, ±hemorrhage, ±vasculitis
Grade 4	Severe ACR Diffuse, polymorphous infiltrate with extensive myocyte damage, ±edema, ±hemorrhage, +vasculitis		

**Table 2:** 2013-ISHLT classification for pathologic diagnosis of AMR.

Grade	Description	Histopathological Findings
pAMR 0	Absence of pathologic AMR	Both histologic and immunopathologic studies negative
pAMR 1 (H+)	Histopathologic AMR alone	Histologic findings present and immunopathologic studies negative
pAMR 1 (I+)	Immunopathologic AMR alone	Histologic findings negative and immunopathologic findings positive
pAMR 2	Pathologic AMR	Both histologic and immunopathologic findings present
pAMR 3	Severe pathologic AMR	Histologic findings of interstitial hemorrhage, capillary fragmentation, mixed inflammatory infiltrates, endothelial cell pyknosis, and/or karyorrhexis and marked edema
